# La calcinose scrotale idiopathique

**DOI:** 10.11604/pamj.2013.14.90.2476

**Published:** 2013-03-07

**Authors:** Fadwa El Amrani, Badredine Hassam

**Affiliations:** 1 Service de Dermatologie, CHU Ibn Sina, Université Med V, Souissi, Rabt, Maroc

**Keywords:** Calcinose scrotale, biopsie, histologie, calcifications, Scrotal calcinosis, biopsy, histology, calcifications

## Image en médicine

La calcinose scrotale idiopathique (CSI) est une affection bénigne, rare, qui se manifeste par des nodules indolores, durs, intéressant la peau scrotale, affectant des patients âgés de 20 à 40 ans, avec des limites d’âge entre 9 et 85 ans. Ces nodules augmentent progressivement de taille et aboutissent à de véritables masses tumorales qui s’ulcèrent et laissent sourdre un matériel crayeux. Le bilan lésionnel ne retrouve aucune autre localisation en dehors de l’atteinte scrotale. Le bilan phosphocalcique sanguin et urinaire de même que le taux d’acide urique et de phosphatase alcaline, de la parathormone, la calcitonine et la vitamine D sont normaux. L’histologie confirme le diagnostic. Le traitement chirurgical est curatif et habituellement il n’y a pas de récidive. Nous rapportons le cas d’un patient de 76 ans, qui consulte pour des nodules scrotaux indolores apparus depuis vingt ans. Ces lésions ont augmenté progressivement de taille et donnaient issue à un matériel blanchâtre crayeux. L’examen retrouvait de multiples nodules scrotaux de couleur jaunâtre, confluant pour former de véritables masses tumorales. Ils étaient durs et mobiles par rapport aux plans profonds. Le reste de l’examen était sans particularité. Le bilan phosphocalcique, l’urée, la créatinine, l’acide urique et les phosphatases alcalines étaient normaux. L’étude anatomopathologique d’une biopsie cutanée montrait des dépôts de basophiles amorphes sièges de calcifications, entourés d’histiocytes et de cellules géantes de type corps étranger confirmant ainsi le diagnostic de CSI. Une exérèse chirurgicale a été réalisée et aucune récidive n’a été notée avec 4 ans de recul.

**Figure 1:**
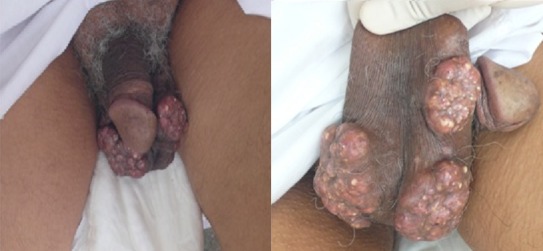
Multiples nodules scrotaux de couleur jaunâtre confluant pour former de véritables masses tumorales

